# Supplementary material for: Manipulation with Mutational Status of VHL Regulates Hypoxic Metabolism and Pro-Angiogenic Phenotypes in ccRCC Caki-1 Cells
Source: Int J Mol Sci. 2025 Oct 31;26(21):10629. doi: 10.3390/ijms262110629 (PMC12608846; doi:10.3390/ijms262110629)
Supplement: Supplementary file 1 [file ijms-26-10629-s001.zip › Supplementary Figure S1.pdf]

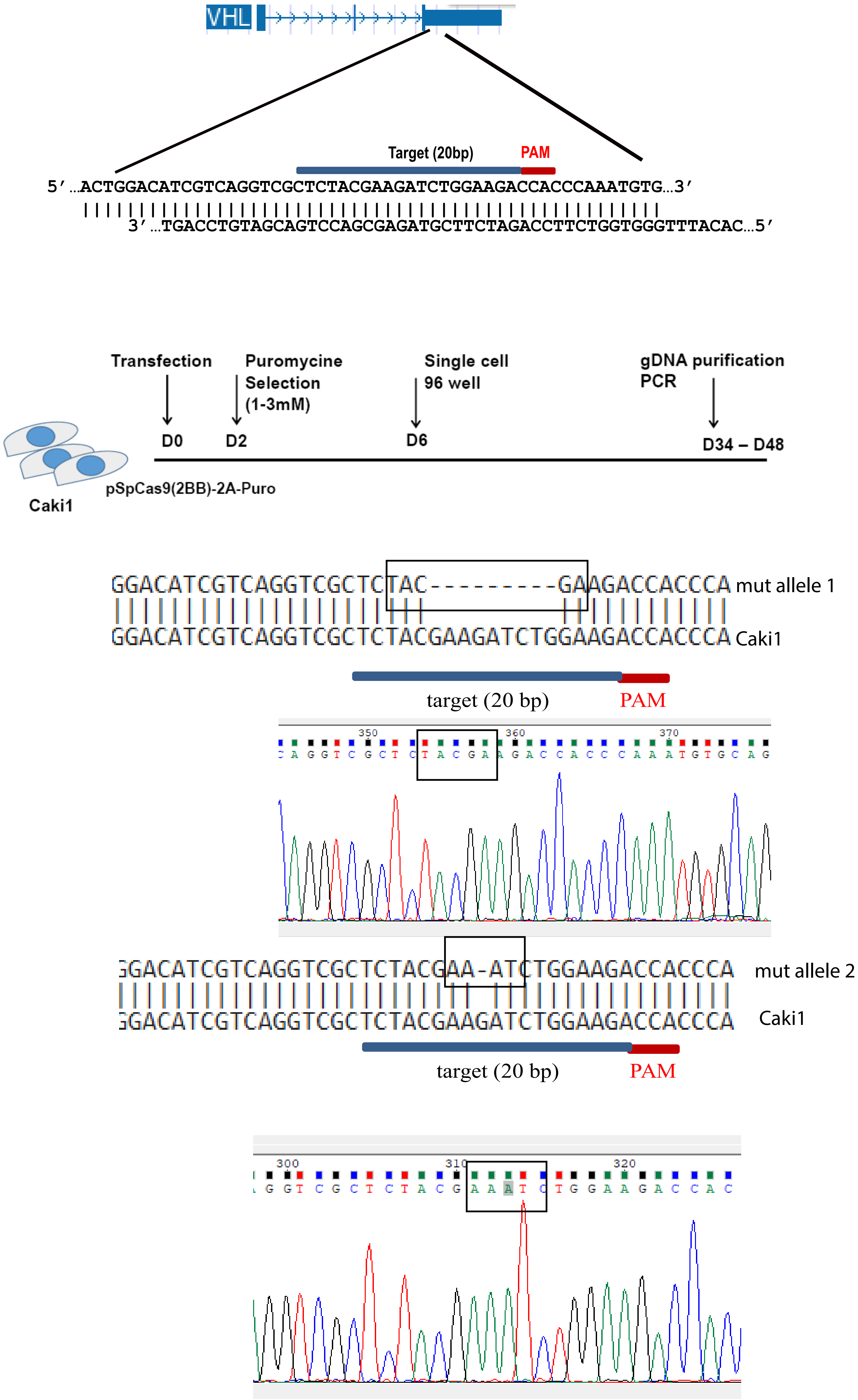

VHL
Target (20bp)
PAM
5′ ...ACTGGACATCGTCAGGTCGCTCTACGAAGATCTGGAAGACCACCCAAATGTG...3′
3′ ...TGACCTGTAGCAGTCCAGCGAGATGCTTCTAGACCTTCTGGTGGGTTTACAC...5′
Transfection
Puromycine Selection (1-3mM)
Single cell 96 well
gDNA purification PCR
D0
D2
D6
D34 – D48
Caki1
pSpCas9(2BB)-2A-Puro
GGACATCGTCAGGTCGCTCTAC---------GAAGACCACCCA mut allele 1
GGACATCGTCAGGTCGCTCTACGAAGATCTGGAAGACCACCCA Caki1
target (20 bp)
PAM
350
360
370
GGACATCGTCAGGTCGCTCTACGAA-ATCTGGAAGACCACCCA mut allele 2
GGACATCGTCAGGTCGCTCTACGAAGATCTGGAAGACCACCCA Caki1
target (20 bp)
PAM
300
310
320
