# Supplementary figures and images for: Manipulation with Mutational Status of VHL Regulates Hypoxic Metabolism and Pro-Angiogenic Phenotypes in ccRCC Caki-1 Cells
Source: Int J Mol Sci. 2025 Oct 31;26(21):10629. doi: 10.3390/ijms262110629 (PMC12608846; doi:10.3390/ijms262110629)

A

DEGs

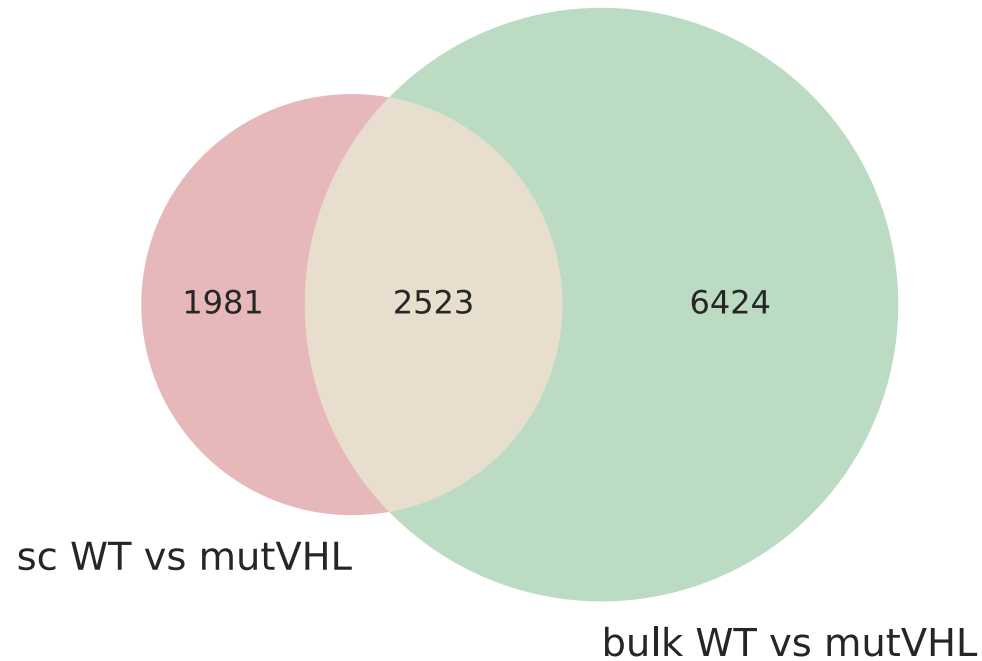

B

DEGs

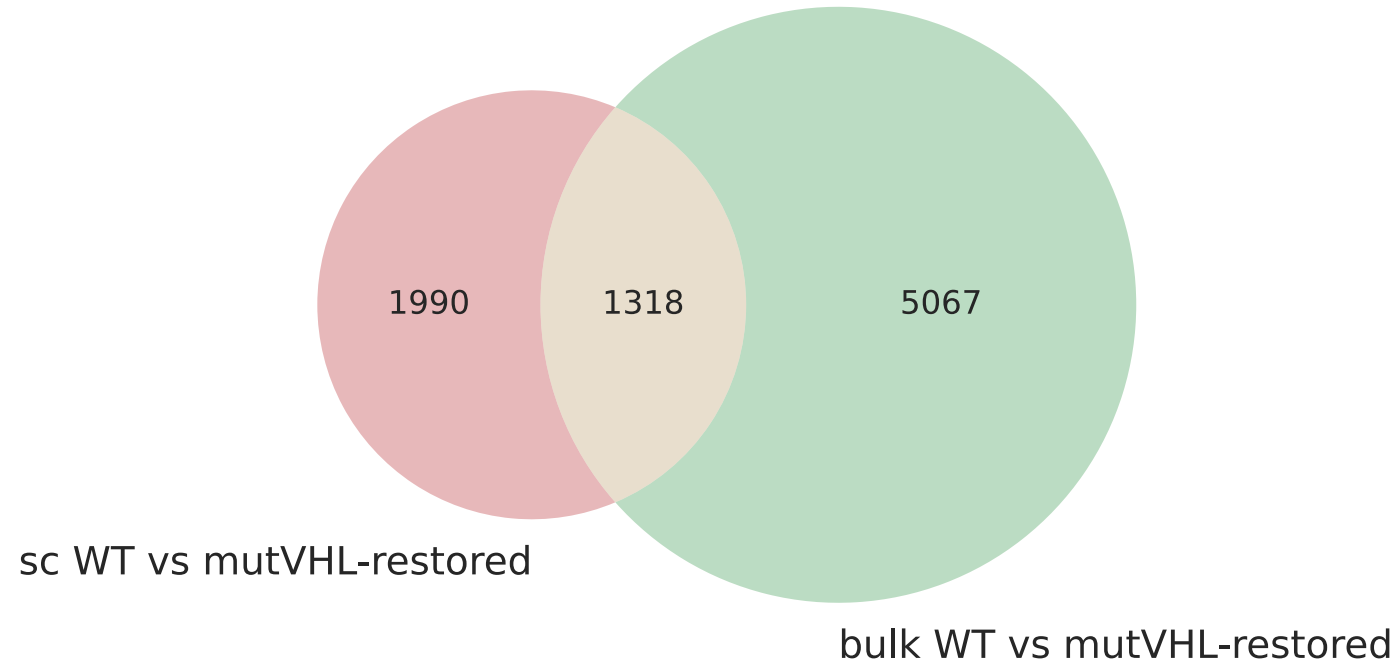

Supplement: Supplementary file 1 [file ijms-26-10629-s001.zip › Supplementary Figure S3.pdf]
